# Dataset of macrobenthic species, organic matter content and grain-size distribution in surficial seafloor sediments in outer continental shelf, pockmark fields and Capbreton Canyon tributaries in the southeastern Bay of Biscay

**DOI:** 10.1016/j.dib.2021.107464

**Published:** 2021-10-09

**Authors:** José Germán Rodríguez, Joxe Mikel Garmendia, Iñigo Muxika, Iñaki Quincoces, Ibon Galparsoro

**Affiliations:** a AZTI, Marine Research, Basque Research and Technology Alliance (BRTA)^1^, Herrera Kaia, Portualdea z/g, Pasaia 20110, Spain; b AZTI, Marine Research, Basque Research and Technology Alliance (BRTA), Txatxarramendi ugartea z/g, Sukarrieta 48395, Spain

**Keywords:** Macrobenthos, Soft-bottom macroinfauna, Continental shelf, Capbreton Canyon, Pockmark fields, Kostarrenkala

## Abstract

This dataset presents the macrobenthic species abundance and biomass in soft bottom areas located at southeastern Bay of Biscay. Data on organic matter content and grain-size distribution in surficial seafloor sediments is also provided. Samples were obtained with Shipek and Smith-McIntyre type grabs in several surveys carried out between 2010 and 2020, covering a range of depths of 32–2241 m. Abundance and biomass of macrobenthic species are given. This database is useful for (i) research on spatial variability of macrobenthic communities, and (ii) baseline knowledge of species in the area. The research article on these data [1] was published in the journal Regional Studies in Marine Science. Title: Macrofaunal variability in the continental shelf and canyons in the southeastern Bay of Biscay.

**Specifications Table**

**Table:** 

Subject	Biological sciences
Specific subject area	Biodiversity of macrobenthos in sedimentary seabed
Type of data	Table
How data were acquired	Sediment samples were obtained with benthic grabs in 2010, 2011, 2012, 2013, 2019 and 2020. Samples for macrofaunal analysis were sieved by 1 mm mesh size and preserved in formalin. Sediment samples were frozen. Laboratory analyses include identification of macrofaunal taxa with microscope, if needed. Sedimentological analysis was performed for the characterization of particle size and organic matter content.
Data format	Raw
Parameters for data collection	The area was previously classified in abiotic habitats types (based on bathymetry, seafloor types and morphology using data derived from multibeam echosounder records, exposition to wave energy and sediment grab samples). Biological sampling locations were distributed among habitat types and spatial gaps of information. Only soft-bottom seafloor was sampled.
Description of data collection	The samples were taken with Shipek (0.04 m^2^) and Smith-McIntyre (0.1 m^2^) grabs.
Data source location	Region: SE Bay of Biscay. Basque continental shelf and Capbreton Canyon tributaries. Country: Spain. Latitude and longitude for collected samples/data: 43.744–43.315 °N, 3.081–1.907 °W. Years: 2010–2020.
Data accessibility [7]	Repository name: Rodriguez, J German; Garmendia, Joxe Mikel; Muxika, Iñigo; Quincoces, Iñaki; Galparsoro, Ibon (2021), “Dataset of macrobenthic species, organic matter content and grain-size distribution in surficial seafloor sediments in outer continental shelf, pockmark fields and Capbreton canyon tributaries in the southeastern Bay of Biscay”, Mendeley Data, V2 Direct URL to data: 10.17632/hvnzwfvsm7.2
Related research article	**Co-submission:** José Germán Rodríguez, Joxe Mikel Garmendia, Iñigo Muxika, María Gómez-Ballesteros, Iñaki Quincoces, Irene Díez, Beatriz Arrese, Francisco Sánchez and Ibon Galparsoro. Macrofaunal variability in the continental shelf and canyons in the southeastern Bay of Biscay. Regional Studies in Marine Science (2021, 102012, https://doi.org/10.1016/j.rsma.2021.102012).

**Value of the Data**

• The dataset is useful for studies on benthic communities’ ecology in the Basque continental shelf and Capbreton Canyon tributaries’ area, as information on benthic communities in the studied area is scarce.
• The results obtained in this research provide valuable information to be compared with other regions of the world.
• The dataset allows to give a baseline of macrobenthic diversity within the area being useful for studies in marine macroecology or studies in presence of allochthonous species.
• The dataset is useful for researchers in marine benthic ecology, as well as for management agencies and governments.

## 1 Data Description

Here we provide a dataset on macrobenthic species density and biomass in soft bottom seafloor located in the south-eastern Bay of Biscay (east Atlantic Ocean), together with sedimentological data on organic matter content and grain-size distribution of surficial sediments.

Regarding the macrobenthic taxa, the original names provided by the taxonomists (in 2010, 2011, 2012, 2013, 2019 and 2020, which are available in the files “Macrobenthos 2010”, “Macrobenthos 2011”, “Macrobenthos 2012”, “Macrobenthos 2013”, “Macrobenthos 2019” and “Macrobenthos 2020”) and their equivalences in WoRMS [2] (except for some taxa), are provided.

Densities and biomass (dry weight) were calculated per square meter.

Regarding the sediment data, mean grain size, percentage of gravel, sand and mud contents and organic matter content are provided. These data are provided in the file “Sediment Characteristics”.

## 2 Experimental Design, Materials and Methods

**Fig. 1:**
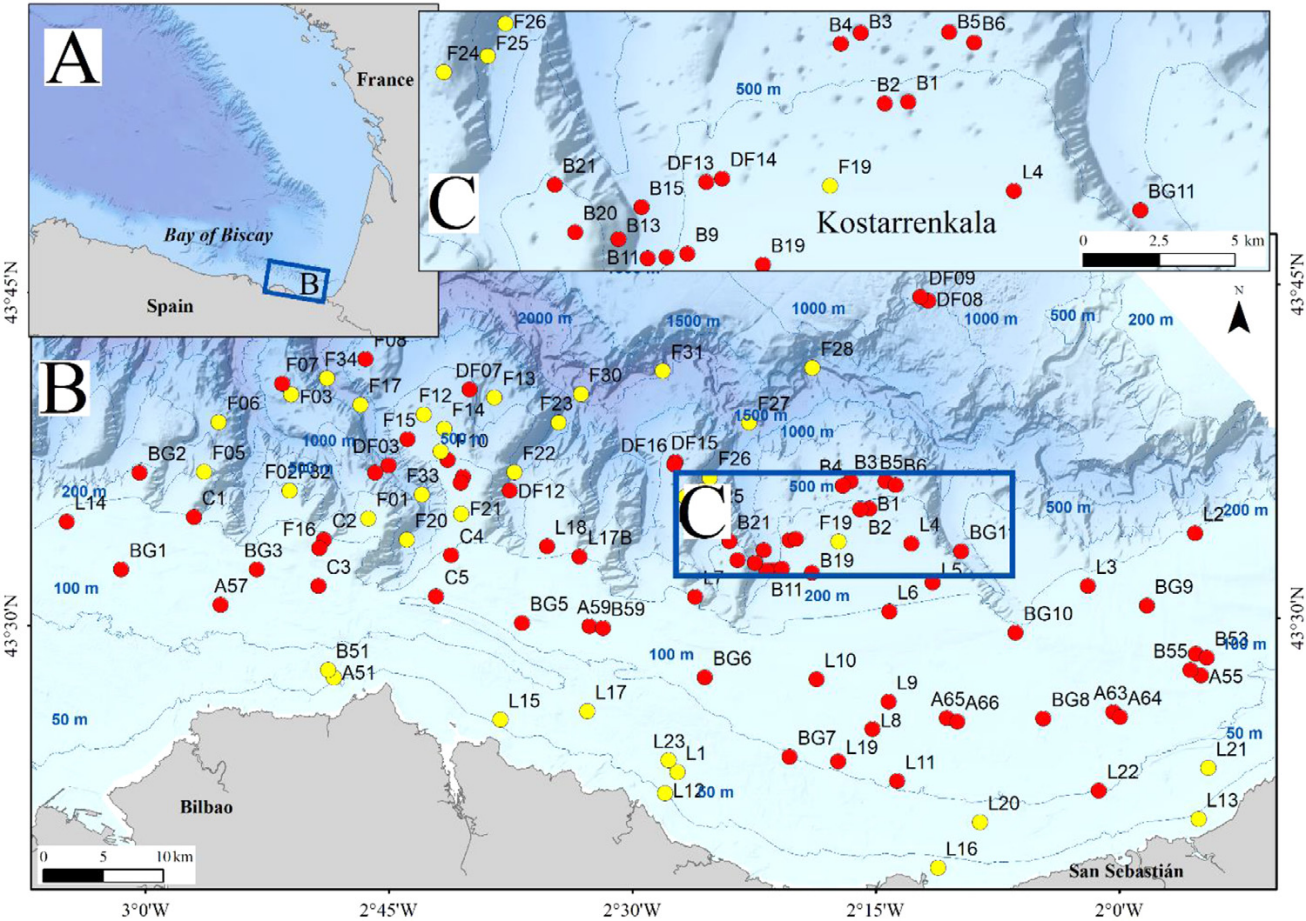
Study area in the SE Bay of Biscay, showing the location of sampling stations. Yellow circles indicate sampling sites sampled with Shipek Grab. Red circles indicate sampling sites sampled with Smith-McIntyre Grab. Modified from Rodríguez et al. [1].

**Fig. 2:**
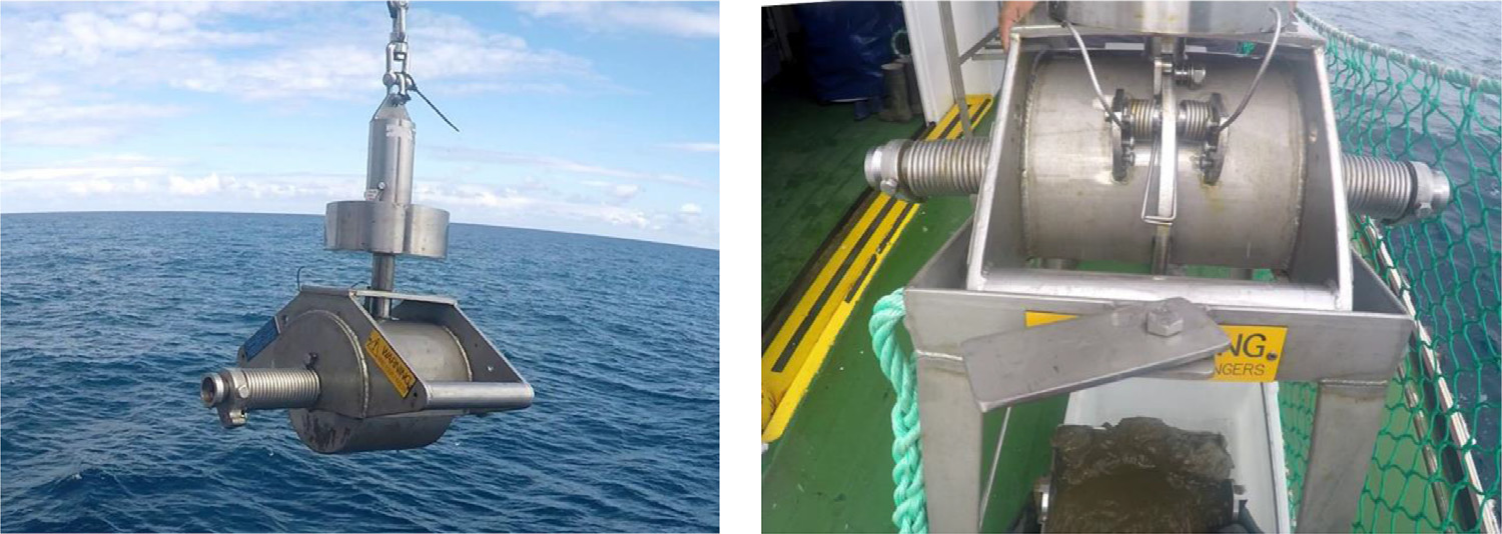
Shipek type grab with 0.04 m^2^ sampling surface.

**Fig. 3:**
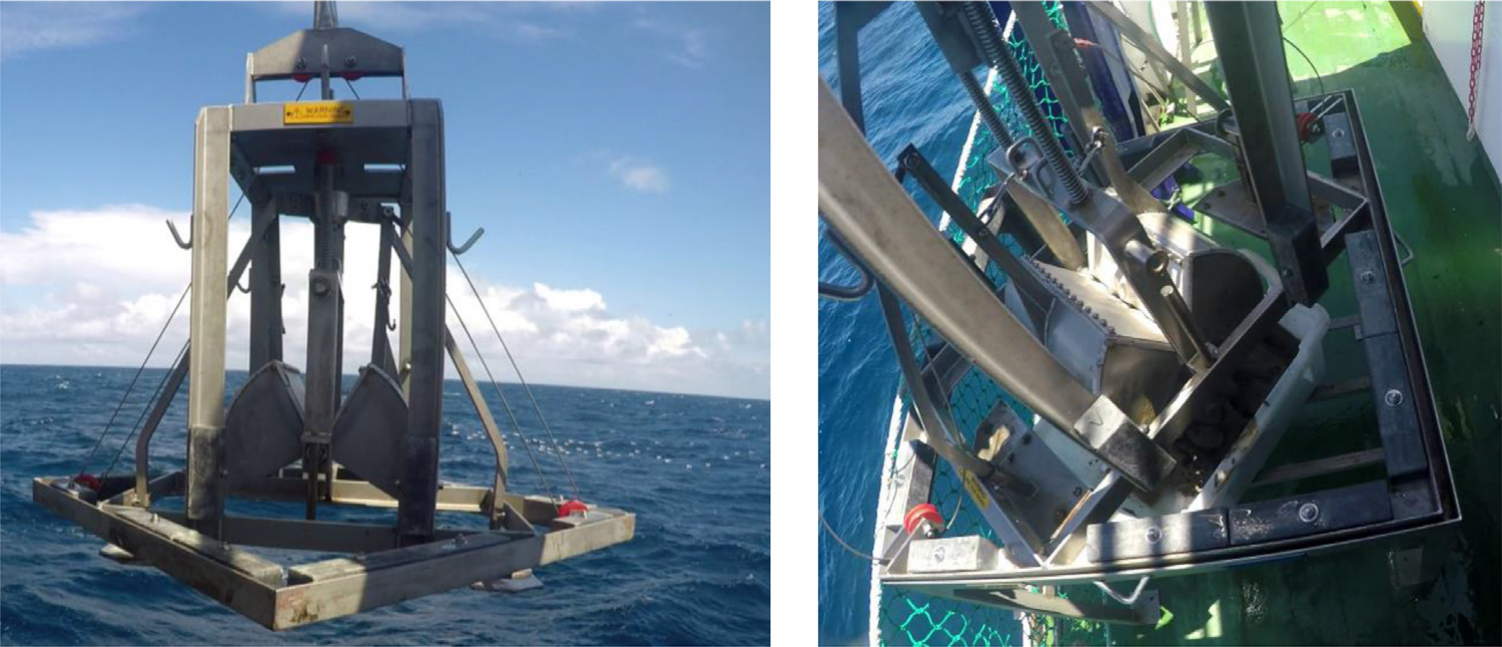
Smith-McIntyre grab with 0.1 m^2^ sampling surface.

**Fig. 4:**
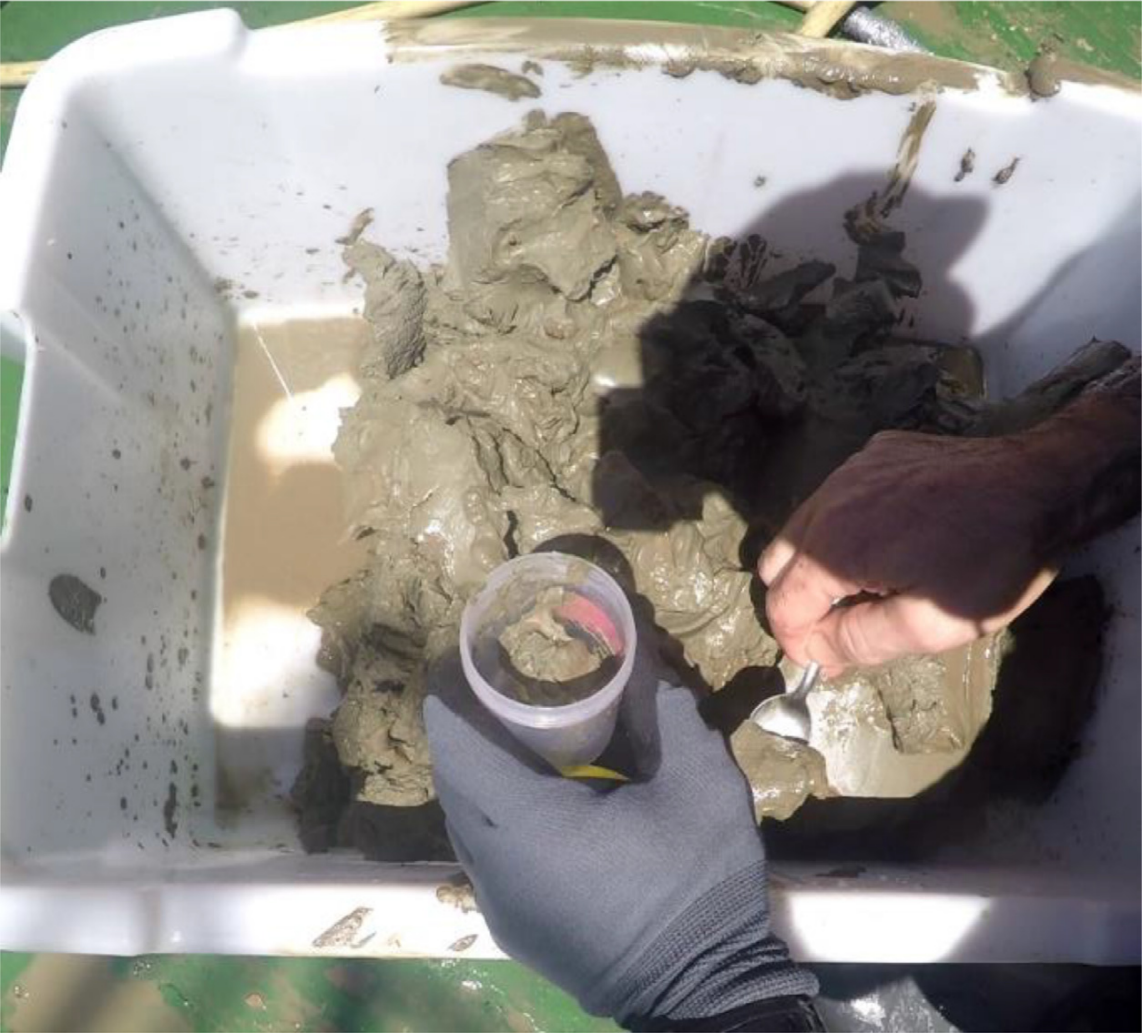
Detail on the subsample for sediment analyses.

**Fig. 5:**
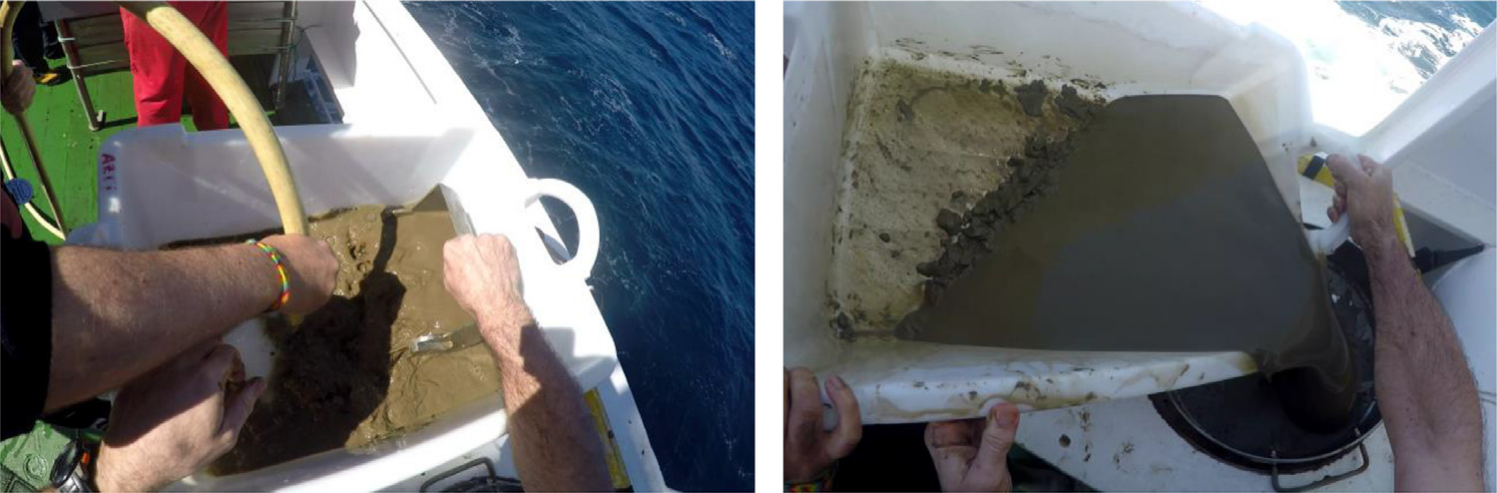
Sieving of sediment through a 1 mm mesh size sieve, for the separation of macrofauna.

A total of 108 sediment samples was sampled in the in the southeastern Bay of Biscay (Basque coast) covering a range of depths of 32–2241 m (Fig. 1). Data were obtained in sampling campaigns carried out in 2010, 2011, 2012, 2013, 2019 and 2020 within the research projects “Demersal ecosystem campaign of the Basque platform with special attention to Capbreton” (June–July 2010), “Research campaign on the fishing ecosystem on the Basque coast” (June–July 2011), “Definition and Development of Ecosystem Management in Basque waters” (June–July 2012 and 2013) and LIFE IP INTEMARES (June–July 2019 and June 2020, https://intemares.es). Sediment samples were taken with a 0.04 m^2^ Shipek (Fig. 2) and 0.1 m^2^ Smith-McIntyre grabs (Fig. 3). For each sample, a subsample of sediment of ca. 140 cm^3^ was extracted and frozen for sediment analysis in laboratory (Fig. 4). The remaining sample was sieved through a 1 mm mesh size (Fig. 5) to extract macrofauna. The sample was preserved in 10% neutralized formalin for laboratory analysis. Although this leads to an effective reduction of the sample, the abundances of macroinfauna were not corrected, and therefore, the densities and biomass were calculated as if the total area sampled was 0.04 m^2^ or 0.1 m^2^ (for Shipek and Smith-McIntyre samples, respectively).

Macroinfauna was identified to the lowest taxonomic level possible and counted by the same group of taxonomists (Sociedad Cultural INSUB, Donostia, Spain) for all the campaigns. Biomass was measured after drying at 90 °C for 24 h.

The particle size distribution of sediments was estimated by (a) dry sieving (only samples with low mud content) or (b) laser diffraction combined with wet sieving of the > 2 mm fraction. The method used for each one was as follows:

a) Dry sieving was carried out using an analytical sieve shaker for 15 min with a set of stainless-steel sieves (mesh sizes: 63, 125, 250, 500, 1000, 2000, and 4000 µm).
b) The laser diffraction particle size analyser (LDPSA) used was a Beckman-Coulter LS 13 320, with a 750 nm laser beam (software version: 4.19). The measurement time adopted was 60 s, with 9–11% of obscuration. Sonication was applied (with a power of 73 W) for 30 s before and during measurement. The Fraunhofer diffraction model was used in the analysis. LDPSA was combined with determination of gravel fraction (i.e., >2 mm) by wet sieving. The results of the measurements done with laser diffraction were transformed into dry sieving equivalent according to Rodríguez and Uriarte [3] as indicated in Table 1.

**Table 1:** Transformations from the results obtained by the laser diffraction particle size analyser to dry sieving equivalent.

Laser diffraction particle size analyser (µm)	Dry sieving equivalent used (µm)
0.375	0.339
4	3.55
6	5.31
8	7.06
11	9.68
16	14.04
22	19.26
31	27.07
44	38.31
63	54.70
90	77.93
125	107.95
180	155.00
250	214.72
355	304.06
500	427.09
710	604.79
1000	849.51
1400	1186.14

Mean grain size was calculated using the graphical method by Folk and Ward [4] with the software GRADISTAT [5]. Percentage of gravel corresponds to the percentage of particles of sediment ≥ 2 mm (in % of weight); the percentage of sand corresponds to the percentage of particles of sediment < 2 mm and ≥ 63 µm (in % of weight or volume); and percentage of mud corresponds to the percentage of particles of sediment < 63 µm (in % of weight or volume).

Organic matter content was calculated by means of weight loss on ignition, at 450 °C during 6 h [6].

## Ethics Statement

The authors declare that they have no known competing financial interests or personal relationships which have, or could be perceived to have, influenced the work reported in this article.

## CRediT authorship contribution statement

**José Germán Rodríguez:** Data curation, Writing – original draft. **Joxe Mikel Garmendia:** Conceptualization, Methodology, Investigation, Writing – review & editing. **Iñigo Muxika:** Investigation, Writing – review & editing. **Iñaki Quincoces:** Conceptualization, Methodology, Resources, Funding acquisition, Writing – review & editing. **Ibon Galparsoro:** Conceptualization, Methodology, Investigation, Writing – review & editing, Supervision, Funding acquisition.

## Declaration of Competing Interest

The authors declare that they have no known competing financial interests or personal relationships which have or could be perceived to have influenced the work reported in this article.
